# Role of the Spore Coat Proteins *CotA* and *CotB*, and the Spore Surface Protein CDIF630_02480, on the Surface Distribution of Exosporium Proteins in *Clostridioides difficile* 630 Spores

**DOI:** 10.3390/microorganisms10101918

**Published:** 2022-09-27

**Authors:** Nicolás Montes-Bravo, Alba Romero-Rodríguez, José García-Yunge, César Medina, Marjorie Pizarro-Guajardo, Daniel Paredes-Sabja

**Affiliations:** 1 ANID—Millennium Science Initiative Program—Millennium Nucleus in the Biology of the Intestinal Microbiota, Santiago 8320000, Chile; 2 Department of Molecular Biology and Biotechnology, Instituto de Investigaciones Biomédicas, Universidad Nacional Autónoma de México, Ciudad de México 34955, Mexico; 3 Department of Biology, Texas A&M University, College Station, TX 77843, USA

**Keywords:** *C. difficile* spores, spore coat, *cotA*, *cotB*, CDIF630_02480, exosporium, CdeA, CdeC, CdeM, BclA

## Abstract

*Clostridioides difficile* is Gram-positive spore-former bacterium and the leading cause of nosocomial antibiotic-associated diarrhea. During disease, *C. difficile* forms metabolically dormant spores that persist in the host and contribute to recurrence of the disease. The outermost surface of *C. difficile* spores, termed the exosporium, plays an essential role in interactions with host surfaces and the immune system. The main exosporium proteins identified to date include three orthologues of the BclA family of collagen-like proteins, and three cysteine-rich proteins. However, how the underlying spore coat influences exosporium assembly remains unclear. In this work, we explore the contribution of spore coat proteins *cotA* and *cotB*, and the spore surface protein, CDIF630_02480, to the exosporium ultrastructure, formation of the polar appendage and the surface accessibility of exosporium proteins. Transmission electron micrographs of spores of insertional inactivation mutants demonstrate that while *cotB* contributes to the formation of thick-exosporium spores, *cotA* and CDIF630_02480 contribute to maintain proper thickness of the spore coat and exosporium layers, respectively. The effect of the absence of *cotA*, *cotB* and CDIF630_02480 on the surface accessibility of the exosporium proteins CdeA, CdeC, CdeM, BclA2 and BclA3 to antibodies was affected by the presence of the spore appendage, suggesting that different mechanisms of assembly of the exosporium layer might be implicated in each spore phenotype. Collectively, this work contributes to our understanding of the associations between spore coat and exosporium proteins, and how these associations affect the assembly of the spore outer layers. These results have implications for the development of anti-infecting agents targeting *C. difficile* spores.

## 1. Introduction

*Clostridioides difficile* is a Gram-positive, anaerobic pathogen. It is the most common cause of nosocomial antibiotic-associated diarrhea worldwide [1,2]. Antibiotic therapy and healthy microbiota disruption are the primary risk factors for *C. difficile* infection (CDI) [1,2]. The main clinical manifestation is diarrhea, but in severe cases, it can produce pseudomembranous colitis, toxic megacolon and death [3]. The main CDI treatment includes metronidazole and/or vancomycin (depending on the disease’s severity) [1,2]. The main complication of CDI is the high recurrence rates of the infection, affecting 20–30% of the infected patients [1,2,3].

During infection, *C. difficile produces* spores, which are highly resistant to diverse factors such as radiation, chlorine-free cleaners and enzymatic activity [4,5]. Furthermore, spore formation is essential for the recurrence of the infection [6], and the spore surface exhibits ligands for specific binding to extracellular matrix proteins in a dose-dependent manner [7], which contributes to *C. difficile* spore-persistence and disease recurrence [8].

The outermost layers of *C. difficile* spores consist of a proteinaceous layer named the spore coat, which is surrounded by an outermost layer named the exosporium [5,9]. The assembly of the spore coat and exosporium layers have features that resemble the formation of membrane-less compartments, as described in eukaryotic cells. The principles that rule the formation of membrane-less compartments occur via phase separation, a process that involves intrinsically disordered regions of Ser, Lys and Arg rich-proteins that act as drivers of the assembly of the layers [10,11,12]. Notably, intrinsically disordered regions are found in spore coat and exosporium proteins of both *B. subtilis* and *C. difficile* [13,14,15]. This suggests that during coat assembly, some of the chemical principles governing the assembly of membrane-less organelles may also apply to spore coat and exosporium assembly [13]. However, knowledge of the underlying mechanism of assembly of the spore coat and exosporium layers is required prior to validating the principle of membrane-less assembly.

The exosporium layer of *C. difficile* spores is described as an electron-dense layer surrounding the spore coat. In most epidemically relevant strains, it is surrounded by hair-like projections [5,9]. Remarkably, clonal populations of *C. difficile* sporulating cultures produce, simultaneously, spores with two distinctive morphotypes: an exosporium layer with a thin- or a thick-electron-dense layer; both morphotypes exhibit hair-like protection [16,17,18]. Among the most prominent proteins of the exosporium layer are three cysteine-rich proteins (CdeA, CdeC, and CdeM), of which at least CdeC and CdeM are implicated in the formation of the exosporium layer [14,19,20,21,22].

In *B. subtilis*, the spore coat is formed by ~80 proteins [23] that form a series of concentric proteinaceous shells around the forespore, similar to *C. difficile* [5]. Despite the ultrastructural similarities between *C. difficile* and *B. subtilis* spores, ~25% of these proteins have homologs in *C. difficile* [24], suggesting that although a similar mechanism might govern the assembly of layers in both species, the proteins implicated are highly divergent [9]. The spore coat proteins in *C. difficile* are currently formed by *cotA*, *cotB*, CotCB, CotD, CotE, CotF and CotG [25,26], some of which have also been identified in the spore exosporeome (i.e., *cotA*, *cotB*, CotCB, CotD, and CotE) [14]. Most of the nomenclature does not correlate with that of *B. subtilis* due to lack of homology, generating confusion in the community.

For example, the *C. difficile* protein named *cotA*, while present in species belonging to the *Clostridioides* and *Paeniclostridium* genera, is missing in *B. subtilis*, highlighting the remarkable differences between these two species. Unlike the *B. subtilis* *cotA*, *C. difficile cotA* has no conserved domain, suggesting no enzymatic activity, nor the ability to bind substrates (metals, cofactors). Notably, *C. difficile* *cotA* is a 35 kDa spore-surface protein, and absence of *cotA* expression leads to the formation of two spore morphotypes, ill-formed spores, with defects in the assembly of the outer layers, and spores with normal ultrastructural features [25], suggesting that *cotA* is essential in stabilizing the formation of the spore coat in a fraction of the spores. Similarly, The *B. subtilis* *cotB* is present as a *cotB/cotH/cotG* cluster which is found in several species closely related to *Geobacillus* [13,27]; however, no *cotB* homologs are present in the *C. difficile* genome [26]. The *cotB* encoded protein in *C. difficile* has a similar molecular weight of 35 kDa to the *B. subtilis* *cotB*. However, it shares no homology with *B. subtilis* *cotB*. The absence of *C. difficile* *cotB* leads to the formation of *C. difficile* spores similar to the wildtype and with a slight decrease in heat resistance, yet it has no impact on the abundance of other spore coat proteins [25].

The forces that drive the formation and shaping of the spore coat and exosporium layer remain unclear. Consequently, in this work, we explored the impact of the spore coat proteins *cotA* and *cotB* on the spore’s ultrastructure and surface distribution of exosporium proteins BclA2, BclA3, CdeA, CdeC, and CdeM in *C. difficile* spores. We also explored the effect of the insertional inactivation of a previously uncharacterized exosporium protein [14], also identified earlier in the spore proteome [28]. This protein-encoding gene was previously annotated as CD630_CD2245.1 in the 630 strain reference genome CD630_AM180355.1 [29] and as CDIFF_02480 in the 630 strain reference genome CD630_CP010905.2 [30], which will be referred to as CDIF630_02480 hereafter. Overall, the results derived from this work provide insights into potential interactions between these constituents and their impact in *C. difficile* spores’ ultrastructural phenotypes.

## 2. Material and Methods

### 2.1. Bacterial Strains and Growth Conditions

*C. difficile* strains (Table 1) were grown under anaerobic conditions using a gas mixture containing 90% N_2_, 5% CO_2_, 5% H_2_. Culture medium (BHIS) was 3.7% brain heart infusion supplemented with 0.5% yeast extract and 1% cysteine broth or on BHIS 1.5% agar plates. The medium was supplemented with the corresponding antibiotics (cefoxitin 10 mg/mL, cycloserine 100 mg/mL, erythromycin 10mg/mL, thiamphenicol 5 mg/mL), depending on the strain. The *C. difficile* strains and plasmids used in this study are summarized in Supplementary Table S1. Standard conjugation techniques were performed to generate the working strains carrying the translational FLAG fusions.

### 2.2. Whole-Genome Sequencing and Mutant Confirmation

Whole-genome sequencing of the mutants generously donated by Dr. Simon Cutting was performed to confirm the presence of the intron and the absence of off-site mutations for all strains used in this study (Table 1). Strains were cultured overnight in 3 mL liquid BHIS, then gDNA was extracted using the Monarch genomic purification kit (NEB, Ispwhich, MA, USA). Library preparation and sequencing was performed by the Microbial Genome Sequencing Center at (MiGS). Briefly, the quantity and quality of DNA were assessed with a Qbit fluorometer and nanodrop, respectively, prior to library preparation using an Illumina DNA Prep kit according to the manufacturer’s protocol. Samples were sequenced on the Illumina NexSeq 2000 platform as paired-end 2 *×* 150 bp reads to generate 200 Mbp total reads.

**Figure 1:**
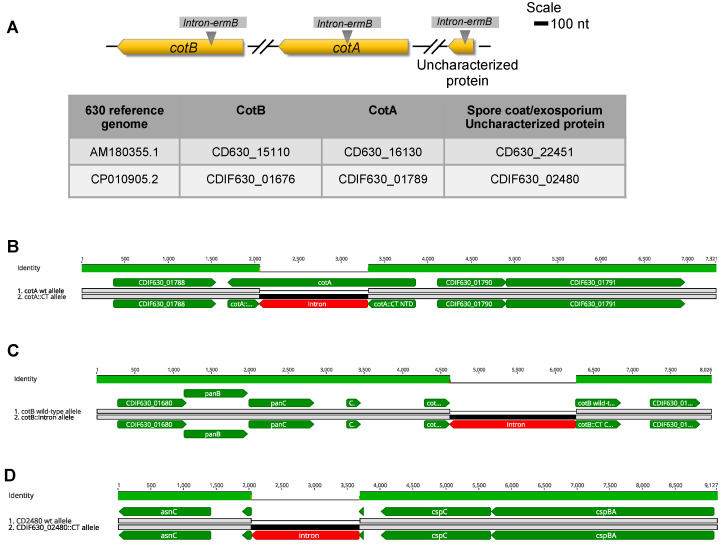
**Analysis of *cotA*, *cotB* and *CDIF630_02480* wildtype and intron-inactivated alleles.** (**A**) Schematic representation of *cotA*, *cotB* and CDIF630_02480 loci in *C. difficile* 630. The table indicates the different codes used in both reference *C. difficile* 630 genomes. (**B**–**D**). Visualization of the pairwise alignment of the *cotA* (**B**), *cotB* (**C**) and CDIF630_02480 (**C**) loci derived from contigs that were de novo assembled with SPAdes, as described in the Methods and Materials section. Green coloring indicates *C. difficile* ORFs native to the loci of interest, whereas the red rectangle indicates the intron. Visualization was made using Geneious Prime software package.

Prior to undergoing de novo assembly of each genome, quality control of reads and adapter trimming were performed using a trimming instrument. For de novo assembly and annotation, SPAdes and RASTtk were used in PATRIC 3.6.9, respectively [31,32,33,34]. Genomes of all strains were aligned to the reference C. difficile 630 genome (CP010905.2) using a Burrows–Wheeler Aligner (BWA-mem), then a single-nucleotide variant and INDELS were analyzed using FreeBayes in PATRIC 3.6.9 [31,35]. Using Clustal Omega [36], the *cotA*, *cotB*, and CDIF630_02480 genes were aligned and presented (Figure 1B–D). Sequenced reads were deposited in the NCBI Sequencing Read Archive under the accession number PRJNA875441.

### 2.3. Plasmid Conjugation

Coat mutant strains were conjugated as described in Diaz et al., 2015, with the plasmids pDP345, pDP360, pDP361, pDP362, pDP365 and pDP369 (Table 1). In short, FLAG-fusion plasmids were transformed into *E. coli* CA434 and subsequently conjugated into *C. difficile* 630Δ*ermB* by co-incubating already transformed *E. coli* CA434 with 200 µL of a *C. difficile* 630Δ*ermB* overnight, which was plated in BHIS agar without antibiotic selection and incubated in anaerobic condition for 7 h at 37 °C. Later, the culture was collected using 500 µL of PBS and plated into supplemented BHIS agar with cycloserine, cefoxitin, and the respective antibiotic selection for each strain for two days in anaerobic conditions at 37 °C. The colonies obtained were isolated two additional times using supplemented BHIS agar plates, as described above, to ensure the presence of the respective plasmid.

### 2.4. Spore Purification

Spores were prepared by plating a 1:1000 dilution of overnight culture onto 70:30 agar plates (63 g Bacto peptone (BD Difco), 3.5 g protease peptone (BD Difco), 0.7 g ammonium sulfate (NH_4_)_2_SO_4_, 1.06 g Tris base, 11.1 g brain heart infusion extract (BD Difco), 1.5 g yeast extract (BD Difco) and 15 g of Bacto agar for 1L). Plates were incubated for seven days at 37 °C in an anaerobic chamber (Bactronez II ^®^, Shellab, OR, USA). After incubation, plates were scraped up with sterile deionized water and washed five times with sterile water at 18,440× *g* for 5 min. The spore suspension was loaded onto a 45% Nycodenz^®^ ((5-(N-2,3-dihydroxy-propyl acetamido)-2,4,6-tri-iodo-N-N′-bis(2,3-dihydroxy propyl) isophthalamide) solution and centrifuged at 18,440× *g* for 45 min. To remove traces of Nycodenz^®^, spores pellets were washed five times at 18,440× *g* for 5 min with sterile water. Finally, spores were counted in a Neubauer chamber, adjusted at 5 × 10^9^ spores per mL and stored in aliquots at −80 °C until use [4]. Three independent spore preparations per *C. difficile* strain were prepared.

### 2.5. Spore Immunofluorescence

First, 5 × 10^7^ spores of *C. difficile* 630*, C. difficile* 630 *cotA*, *C. difficile* 630 *cotB*, *C. difficile* 630 *cotCB* were fixed in poly-L-lysine (Sigma-Aldrich, MA, USA)-coated glass cover slides with paraformaldehyde (pH 7.4) for 20 min. Fixed spores were washed three times with PBS, blocked with BSA 1% for 1 h (Sigma-Aldrich, USA) and incubated with primary antibody 1:250 rabbit anti-FLAG-IgG (Rockland 600-401-383). The cover was washed three times with PBS and incubated for 1 h with secondary antibody 1:500 anti-rabbit IgG-Alexa Fluor 488 conjugated (A32731, Invitrogen, MA, USA) and washed three times with PBS. Once the cover dry, it was mounted with the Dako fluorescence mounting medium (Dako Morth America, CA, USA) and sealed with transparent nail polish. Samples were analyzed with a BX53 Olympus fluorescence microscope. The fluorescence intensity was quantified using ImageJ [37]. Three biological replicates were performed for each *C. difficile* strain–plasmid combination.

### 2.6. Transmission Electron Microscopy

To analyze the ultrastructure of the *C. difficile* 630, *cotA*, *cotB* and *CDIF630_02480* strains, the purified spores were centrifuged at 18,440× *g* for 5 min and fixed in glutaraldehyde 3% and cacodylate buffer (pH 7.4) overnight and stained for 30 min with 1% tannic acid, as described [17,18]. The samples were processed and embedded in spurs resin, as previously described. Thin sections obtained with a microtome were placed on glow discharge carbon-coated grids and double lead stained with 2% uranyl acetate and lead citrate. Grids were analyzed with a Phillips Tecnai 12 Bio Twin at the Electron Microscopy Facility of the Pontificia Universidad Católica de Chile.

To analyze the length of spore layers, transmission electron micrographs of 10 representative spores with thin and thick exosporium were selected as we have described [16,17,18,38]. For each spore, the length of the layers was quantified at six different locations and the mean was represented in the graphs as described [38].

### 2.7. Statistical Analysis

For each cover, at least 200 spores were quantified. The exposition time was the same between the control and the sample. Student’s *t*-test was used for the comparison between the samples, and Mann–Whitney non-parametric test was used for non-parametric samples. All statistical analyses were performed using GraphPad Prism Version 7 for Windows (GraphPad Software, La Jolla, CA, USA).

## 3. Results

### 3.1. In Silico Analysis of CDIF630_02480 and Whole-Genome Analysis of Clotron-Insertional Mutants

Proteomic studies have shown that *cotA*, *cotB* and CDIF630_02480 are present in the exosporium layer of C. difficile 630 spores [14]. Notably, transcriptional studies suggest that both *cotA* and *cotB* are regulated by the early mother-cell-specific RNA polymerase sigma factor SigE, whereas CDIF630_02480 is regulated by SigF [39,40]. CDIF630_02480 ORF encodes a 64 amino acid polypeptide in the antisense strand and is localized 2070 bp downstream of an asparagine-tRNA ligase and 467 bp upstream of the bile salt germinant receptor protein CspC encoding gene (Figure 1A). The predicted MW of the encoded protein is 6.9 kDa, and it exhibits a domain of unknown function (DUF3787 superfamily domain) which is conserved in the Clostridia. Analysis of publicly available genomes in NCBI revealed that CDIF630_02480 is present in all 2261 analyzed draft genomes of *C. difficile*, whereas BlastP analysis in the database of Microbial_proteins reveals that 86% of the hits belong to members of the Peptostreptococcaceae family and the remainder to members of the Clostridiaceae family. These observations suggest that, despite CDIF630_02480 being potentially expressed in the forespore and found in the exosporium, proteome may play a role in the assembly of the spore surface layers.

Previous work characterizing *cotA* and *cotB* mutants, while conducted with rigor, did not ensure that the Clostron-derived intron insertion occurred uniquely in the gene of interest. Consequently, all three mutant strains were whole genome sequenced, and illumina reads trimmed and de novo assembled by SPAdes to provide unbiased assembled contigs, which were annotated and used to identify intron insertion site(s). As a control, we whole-genome sequenced *C. difficile* 630 delta *ermB*. The results demonstrated that the de novo assembled contigs of strains *cotA*, *cotB* and CDIF630_02480 contained a single copy at positions *cotA*::CT555a, *cotB*::CT329a and CDIF630_02480::CT60a (Figure 1B–D and Supplementary Figures S1–S3).

### 3.2. Inactivation of CotA, CotB and CDIF630_02480 Genes Produces Changes in the Ultrastructure of the Spore

**Figure 2:**
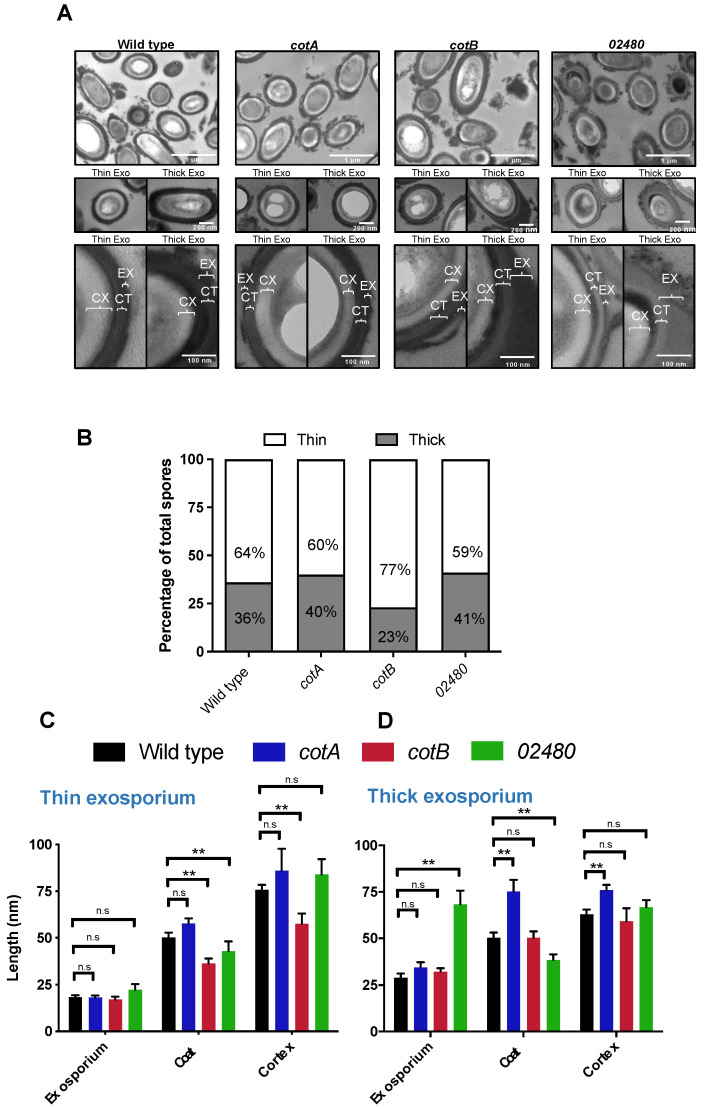
**Effect of absence of spore coat proteins in the ultrastructure of *C. difficile* spore** (**A**). Thin sections of *C. difficile* 630∆*ermB* and coat mutant derivatives *(cotA::CT555a*, *cotB cotB::CT329a* and *CDIF630_02480::CT60a*) spores were analyzed via transmission electron microscopy, as described in the Methods and Materials section. Representative micrograph of several *C. difficile* 630∆*ermB* (wildtype), *cotA::CT555a*, *cotB cotB::CT329a* and *CDIF630_02480::CT60a* spores are shown in the upper panel. The middle panel shows a representative individual spore with a thin and thick exosporium layer of wildtype, *cotA::CT555a*, *cotB cotB::CT329a* and *CDIF630_02480::CT60a*) strains. The lower panel shows a magnified view of spores with thin and thick exosporium layers, indicated as EX—exosporium; CT—coat; and CX—cortex. (**B**) Effect of coat proteins mutation in the exosporium thickness. The exosporium thickness of wildtype, *cotA::CT555a*, *cotB::CT329a* and *CDIF630_02480::CT60a* spore was analyzed for at least 62 individual spores. (**C**,**D**) The thickness of the exosporium and outer and inner coat layers of *C. difficile* wildtype (black), *cotA::CT555a* (blue)*,* *cotB::CT329a* (red) and *CDIF630_02480::CT60a* (green) strains were analyzed via transmission electron microscopy of least ten individual spores with an apparent thick (**C**) and thin (**D**) exosporium morphotype. Statistical analysis is One-Way ANOVA followed by Sidak´s multiple comparison test. Error bars denote the standard error of the mean. Asterisks denote statistically significant differences (ns, not significant); **, *p* < 0.01.

The spore coat *cotA* and *cotB* mutants have been previously characterized [25]; however, a thorough analysis of the spore´s ultrastructural properties was not achieved (i.e., ratio of thick- and thin-exosporium spores, and thickness of the cortex, spore coat and exosporium layer). Moreover, the role of CDIF630_02480 (Table 1) on *C. difficile* spore biology and assembly of the outer layers remains unknown. Here, we sought to quantify the impact of insertional inactivation of *cotA*, *cotB*, and *CDIF630_02480* genes on the *C. difficile* spores’ ultrastructural properties. For these purposes, we produced spores from strains containing insertional inactivation of *cotA*, *cotB* or *CDIF630_02480* genes in 70:30 agar plates, and examined purified spores using transmission electron microscopy (TEM) to assess the ultrastructural changes. Notably, although previous work suggests that *cotA* had a major role in spore assembly when *cotA* spores were prepared in liquid BHI broth, our results demonstrate that *cotA* spores had no major structural defects (Figure 2A), whereas *cotB* and *CDIF630_02480* spores had an overall appearance similar to wildtype spores (Figure 2A). Despite the ultrastructural similarities between *cotA*, *cotB* and *CDIF630_02480* and wildtype spores, some slight variations in the thickness of the outer spore layers were evident (see below).

A major feature of the exosporium layer in *C. difficile* is the formation of two distinctive exosporium morphotypes from clonal population, spores with a thick- or a thin-exosporium layer [16,17,18]. This phenotype has been observed in strain 630, which lacks the hair-like projections observed in all analyzed clinically relevant strains to date, thus rendering 630 spores with a thick- or thin-smooth electron-dense exosporium layer [5,17,18]. The mechanisms driving the formation of these morphotypes remain unknown; therefore, we evaluated whether inactivation of the coat proteins, *cotA* and *CDIF630_02480* and *cotB* would affect the ratio with which both exosporium morphotypes are formed. Similarly, as we have previously reported, wildtype strain 630 formed spores where 64% of the spores had a thin exosporium, while 36% of the spores presented the thick exosporium. Analysis of *cotA* and *CDIF630_02480* spores revealed no significant changes in the percentages of thick (60 and 59%, respectively) or thin exosporium (40 and 41%, respectively) compared to the parental strain. However, the *cotB* mutant produced fewer thick-exosporium spores (23%) and a higher percentage of thin-exosporium spores (77%) (Figure 2B). This result suggests that *cotB*, but not *cotA* and CDIF630_02480, contributes to the formation of thick-exosporium spores.

### 3.3. Inactivation of CotA, CotB and CDIF630_02480 Genes Alters Spore Layers Thickness

To gain insight into how the absence of the coat proteins affects the spore structure, the length of spore layers cortex, coat and exosporium were measured. For this, TEM images were classified as thick or thin exosporium, as we have previously described [16,17,18], and later, the length of each layer was measured (Figure 2C). Spores of *cotA* mutant with a thin exosporium did not show significant changes in the length of exosporium, coat or cortex compared with the wildtype strain (Figure 1C). Conversely*,* thick-exosporium *cotA* spores showed an increase in the thickness of the coat of approximately 25 nm (from 50 nm in the parental strain to 75 nm in the *cotA* mutant) (Figure 2C). The cortex’s length in the *cotA* mutant was longer than the wildtype strain (76 nm and 62 nm, respectively) (Figure 2C). For thick-exosporium *cotB* spores, no significant changes were observed in the length of the exosporium, coat or cortex layers (Figure 2C). Contrarily, thin-exosporium *cotB* spores showed significant changes in the coat and cortex’s length (Figure 2D). The coat length of the *cotB* mutants was lower (36 nm) than that presented in the parental strain (50 nm). In thick-exosporium *CDIF630_02480* spores, a significant difference (*p* < 0.01) was found in the length of the exosporium and coat (Figure 2D). The exosporium length in the *CDIF630_02480* mutant was almost 2.5 times thicker than in the parental strain (68 nm and 29 nm, respectively). On the contrary, the coat’s length was 12 nm smaller in the *CDIF630_02480* mutant than in the parental strain (38 nm and 50 nm, respectively) (Figure 2D). Additionally, the coat of thin exosporium *CDIF630_02480* spores was thinner than the wildtype (42 nm and 50 nm, respectively) (Figure 2D). In summary, these results demonstrate that in thin-exosporium spores, the absence of *cotB* and CDIF630_02480 leads to a thinner coat layer, whereas in thick-exosporium spores, the absence of *cotA* and CDIF630_02480 leads to thickening of the coat and exosporium layers, respectively.

### 3.4. CotA, CotB and CDIF630_02480 Proteins in Spore Appendage

**Figure 3:**
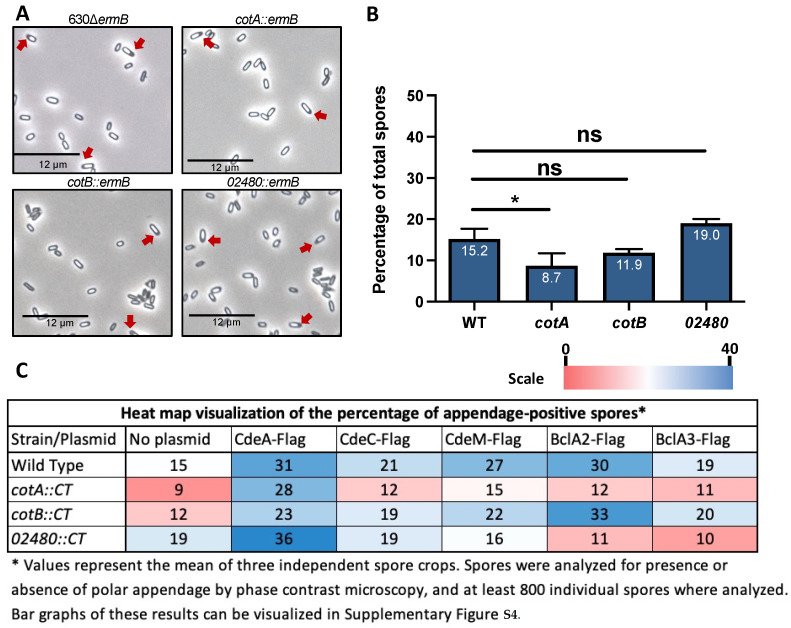
**Effect of absence of coat proteins in the spore appendage of *C. difficile* spores.** Spores of wildtype, *cotA::CT555a*, *cotB::CT329a* and *CDIF630_02480::CT60a* strains were purified, mounted in glass coverslips and analyzed via phase-contrast microscopy, as described in the Materials and Methods section (**A**) Representative micrograph of wildtype, *cotA::CT555a*, *cotB::CT329a* and *CDIF630_02480::CT60a* spores, where the reds arrows show the presence of spores with an appendage in the spore population of each strain. (**B**) The spores with an appendage (blue bars) or without an appendage (difference to total spores) in each strain’s spore population were analyzed via a phase-contrast micrograph; at least 800 spores were analyzed. Asterisks denote statistically significant differences relative to the control wildtype (ns, not significant; *, *p* < 0.05). Statistical analysis is One-Way ANOVA followed by Sidak´s multiple comparison test. (**C**) Effect of absence of spore coat proteins and overexpression of exosporium proteins in the formation of polar appendage in *C. difficile* spores. Purified spores were analyzed via phase-contrast microscopy, as described in the Methods and Materials section. *C. difficile* strains carrying plasmid pMTL82151 with CdeA-Flag, CdeC-Flag, CdeM-Flag, BclA2-Flag and BclA3-Flag fusions. The spores with an appendage in each strain’s spore population were analyzed via a phase-contrast micrograph; at least 800 spores were quantified. Data are a heat map visualization of the percentage of appendage-positive spores. Original data with statistical differences are presented in Supplementary Figure S4.

Another structural feature that has been recently reported in *C. difficile* spores [16,21] is that a small fraction of spores present a terminal appendage on one of their poles. The mechanism underlying pole formation remains unclear. Consequently, since inactivation of *cotA*, *cotB* and *CDIF630_02480* genes affected the length of the spore layers, we asked whether these genes also played a role in the presence of a polar appendage. Phase contrast microscopy analysis of wildtype and mutant spores revealed that while inactivation of *cotB* and *CDIF630_02480* had no significant effect on the presence of a polar appendage, inactivation of *cotA* led to a significant decrease from ~ 15 to ~9% of the spores with a polar appendage (Figure 3A,B), suggesting that *cotA*, but not *cotB* and CDIF630_02480, contributes to the formation of polar appendages.

We have recently reported that expression of exosporium and spore coat proteins SNAP-tag reporter fusions under the control of their native promoter, using pMTL multi-copy vectors in the epidemically relevant R20291 wildtype strain, leads to changes in the abundance of appendage-bearing spores [16]. This was a consequence, in part, of the use of multicopy pMTL series vectors that increase the copy number of the gene of interest [41], leading to increased expression and consequent changes in the titration of spore coat and exosporium constituents during spore assembly [42]. Therefore, here, we took advantage of the smaller molecular mass of the Flag epitope, which was fused to selected exosporium proteins to address how overexpression of each individual exosporium protein impacts the presence of appendages in each spore coat mutant genetic background (Figure 3C). First, we focused on the three cysteine-rich exosporium proteins, CdeA, CdeC and CdeM; of which CdeC and CdeM have been reported to be essential for the assembly of the outermost layer [19,20], while the role of CdeA remains unknown. Notably, overexpression of CdeA in a wildtype strain led to a two-fold increase in the abundance of polar appendage (~31% of total spores) compared to wildtype spores (~15% of total spores) (Figure 3C and Figure S4A). Upon overexpressing CdeA, an absence of CDIF630_02480, but not *cotA* or *cotB*, led to a slight increase in appendage-bearing spores from 31 to 36% (Figure 3C and Figure S4A). Increased expression of the CdeC led to a slight, but not significant increase in appendage-positive spores in a wildtype background; however, an absence of *cotA*, *cotB* or CDIF630_02480 led to no significant changes in the abundance of appendage-positive spores (Figure 3C and Figure S4B). In contrast with CdeC, overexpression of CdeM led to substantial changes in the abundance of appendage-positive spores (Figure 3C and Figure S4C); upon increased expression of CdeM in wildtype strain, appendage-positive spores significantly increased to ~27% of total spores when compared to wildtype spores (Figure 3C and Figure S4C). These high levels of appendage-positive spores are also maintained when overexpressing in a *cotB* mutant, but they decrease to wildtype levels in the absence of *cotA* and CDIF630_02480 (Figure 3C and Figure S4C). Taken together, these results suggest that among the cysteine-rich proteins, increased expression of CdeA, regardless of the genetic background, positively contributes to appendage formation. Meanwhile, overexpression of CdeM contributes to appendage formation, which is strengthened by the presence of *cotA* or CDIF630_02480.

Most *C. difficile* strains, such as 630, encode three orthologues of the BclA family of proteins (i.e., BclA1, BclA2 and BclA3) [43,44]; however, it is unclear how the titration of these proteins could affect morphological spore properties, specifically the polar spore appendage. Unfortunately, although we attempted to produce spores carrying each of the BclA proteins, we were unable to obtain spore preparations of the coat mutant strains carrying BclA1-FLAG fusion; therefore, we conducted this analysis with strains overexpressing BclA2 and BclA3 (Figure 3C and Figure S4D,E). Overexpression of BclA2 in a multicopy plasmid led to a two-fold increase in the abundance of polar appendages in a wildtype strain; the absence of *cotA* and CDIF630_02480 significantly reduced the formation of a polar appendage upon BclA2 overexpression (Figure 3C and Figure S4D,E). In contrast, overexpression of BclA3 in wildtype strain led to no significant increase in appendage formation, and the absence of *cotA*, *cotB* and CDIF630_02480 had no impact on appendage formation upon overexpressing BclA3 (Figure 3C and Figure S4D,E). In summary, these results suggest that polar appendage is positively influenced by the expression of BclA2, which requires the presence of *cotA* and CDIF630_02480.

### 3.5. Absence of Coat Proteins Differentially Affects the Accessibility of Cysteine-Rich and Collagen-like Proteins to Antibodies

**Figure 4:**
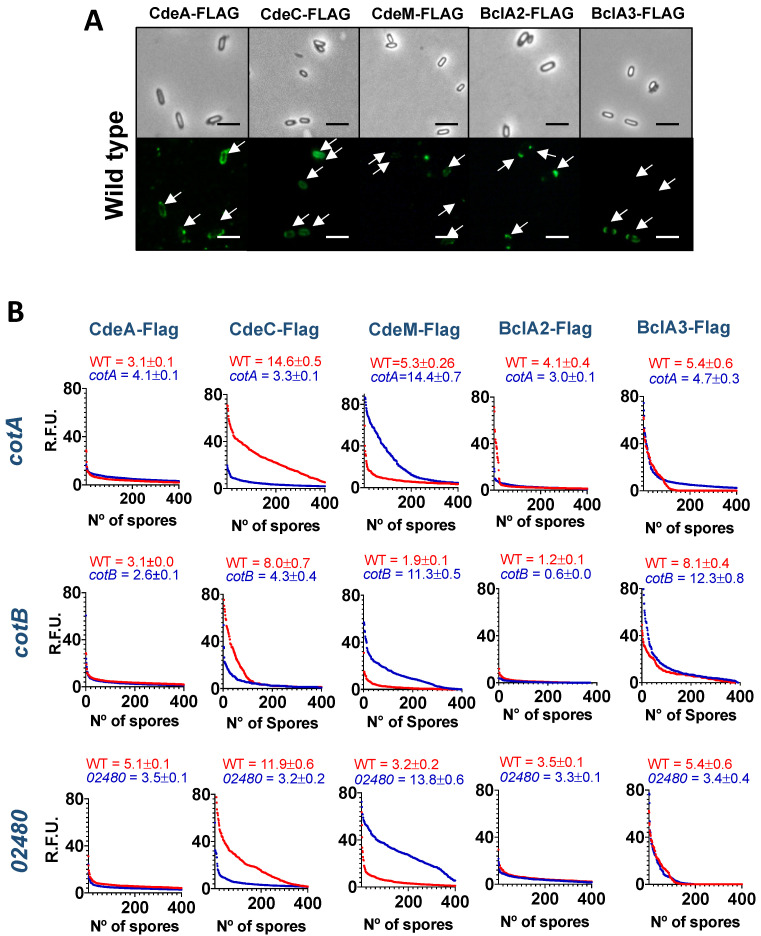
**Effect of absence of spore coat proteins in accessible fluorescence of Flag fusion exosporium proteins.** (**A**) Representative micrographs of accessible surface fluorescence intensity of spore coat and exosporium protein Flag fusions expressed in purified wildtype 630 spores. (**B**) Quantitative analysis of the fluorescence intensity expressed as Relative Fluorescence Units (RFU) of wildtype, *cotA (cotA::CT555a*), *cotB* (*cotB::CT329a*) and *CDIF630_02480* (*CDIF630_02480::CT90a*) mutant strains carrying Flag fusions with spore coat and exosporium proteins stained with anti-FLAG antibodies, as described in Materials and Methods. Graphs represent the counts of 400 spores, which is a representative biological replicate to demonstrate the distribution pattern. The values on top of each graph represent the average and standard error of the mean of the three independent biological replicates.

Having considered how overexpression of exosporium proteins affected the presence of appendages, to gain insight into the association of spore coat and exosporium proteins, we assessed how the absence of selected spore coat proteins would impact the accessibility of cysteine-rich proteins to anti-Flag antibodies. Therefore, we took advantage of previously constructed translational FLAG fusions for these exosporium proteins to address the accessibility to anti-FLAG antibodies [14,20]. Briefly, purified spores of wildtype and mutant strains were assayed by immunofluorescence against the FLAG epitope and analyzed via fluorescence microscopy. Analysis of the fluorescence intensity of CdeA-FLAG fusion-carrying spores revealed that insertional inactivation of *cotA*, *cotB* and *CDIF630_02480* had a differential impact on antibody accessibility to the CdeA-FLAG fusion (Figure 4). While the absence of *cotA* led to a significantly increase in antibody accessibility, the absence of *cotB* and CDIF630_02480 significantly reduced the antibody accessibility of CdeA (Figure 4A). In the case of CdeC-Flag fusion, the absence of all three spore coat proteins led to a significant decrease in fluorescence intensity (Figure 4B), suggesting that *cotA*, *cotB* and CDIF630_02480 are required for CdeC surface accessibility to antibodies. In contrast, the absence of *cotA*, *cotB* and CDIF630_02480 caused an increase in CdeM-Flag-specific fluorescence intensity in all spore coat mutant strains compared to wildtype spores (Figure 4C). These observations suggest that the spore coat proteins *cotA*, *cotB* and CDIF630_02480 might be masking CdeM towards antibody accessibility.

Having observed how the absence of the spore coat proteins *cotA*, *cotB* and CDIF630_02480 affected the surface accessibility of the exosporium cysteine-rich proteins, we extended this analysis to the collagen-like BclA proteins. Analysis of fluorescence micrographs of BclA2-FLAG carrying wildtype and coat mutant spores revealed that although absence of *cotA* and *cotB* led to a significant decrease in BclA2-fluorescence intensity, this decrease was low (Figure 4D). Fluorescence analysis of BclA3-FLAG fusion carrying spores revealed that *cotA* and *CDIF630_02480* spores exhibited significantly lower fluorescence intensity than wildtype spores (Figure 4C). In contrast, a significant increase in fluorescence intensity of BclA3 was observed in *cotB* spores compared to wildtype (Figure 4C). These observations suggest that *cotB* might be covering antibody accessible sites of the BclA3-FLAG fusion.

### 3.6. Effect of Insertional Inactivation of Spore Coat Proteins on the Immunofluorescence Distribution of Cystein-Rich Flag Fusion Proteins in the Presence/Absence of Appendage

**Figure 5:**
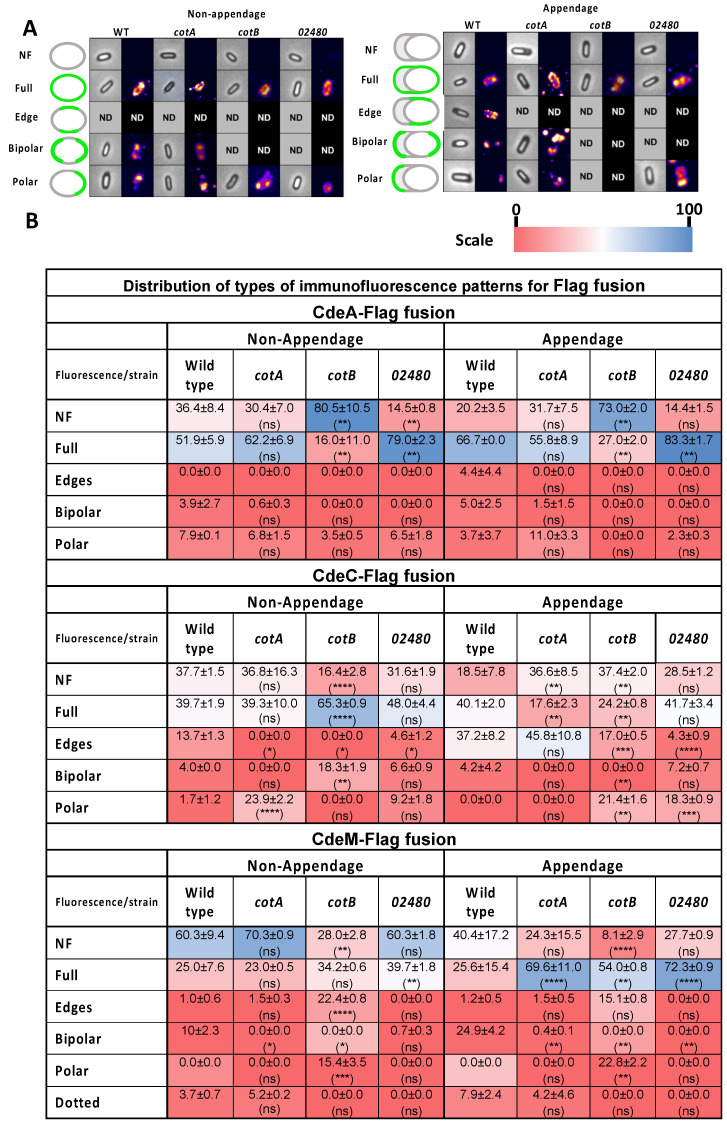
**Effect of absence of spore coat proteins in accessible immunofluorescence of Flag fusions of cysteine-rich exosporium proteins**. (**A**) Representative image of the distribution of fluorescent distribution pattern of CdeA-FLAG on the surface of non-appendage or appendage spores of wildtype and *cotA (cotA::CT555a*), *cotB* (*cotB::CT329a*) and *CDIF630_02480* (*CDIF630_02480::CT90a*) mutant strains. (**B**) Quantification of fluorescence patterns in non-appendage or appendage spores of wildtype *cotA*, *cotB or CDIF630_02480* strains, which were first binned by presence or absence of appendage through phase contrast microscopy prior to fluorescence analysis. Data represents the mean and error bars represent the standard error of the mean. Asterisks denote statistical difference respect to wildtype (ANOVA and Bonferroni multiple comparison test), where * *p* < 0.05, ** *p* < 0.01, *** *p* < 0.001, **** *p* < 0.0001, and n.s., denotes no significant difference. Heat map depicts the percentage of total of the fluorescence distribution from 0 to 100%.

Next, we explored the fluorescence distribution pattern of cysteine-rich FLAG fusion proteins in the different spore coat mutant backgrounds and how these were affected by the presence/absence of the polar appendage. In order to achieve this, patterns of distribution were classified depending on the fluorescence localization within the spore: along the whole spore (denominated as *Full*), distributed mainly in one pole of the spore (named *Polar*), present mainly in both poles of the spore (*Bipolar*) and fluorescence localized in the lateral sides of the spore, not on the poles (*Edge*) (Figure 5B–D and Figure S5A).

In wildtype non-appendage spores, CdeA was distributed primarily as a *Full* pattern (52%) phenotype, while the occurrence of the Polar and Bipolar patterns was detected in 8% and 4%, respectively (Figure 5B). Insertional inactivation of *cotA* and *cotCB* had no major effect on fluorescent patterns of CdeA (Figure 5B). However, a *cotB* mutation led to a substantial shift from full- to non-fluorescence of CdeA (Figure 5B). In wildtype appendage spores, CdeA was mainly distributed along the spore as a *Full* pattern (66%), while 4.4, 5.0 and 3.7% of the spores presented the *Edge*, *Bipolar* or *Polar* patterns, respectively (Figure 4C,D and Figure S5A). As in the appendage’s spores, no major changes in CdeA fluorescence pattern were observed in the absence of *cotA*, while absence of *cotB* led to a shift from full- to non-fluorescence and absence of *CDIF630_02480* led to a slight, but significant increase in full-fluorescence pattern (Figure 5B). Taken together, the main fluorescence phenotype of CdeA is an even distribution on the spore surface, which is not affected by the presence/absence of the polar appendage and requires the presence of *cotB*.

The distribution of CdeC was mainly identified as a *Full* pattern (39.3 ± 10%), while *Edge*, *Polar* and *Bipolar* patterns were less abundant (13.7 ± 1.3%, 1.7 ± 1.2% and 4.0 ± 0.0, respectively). The distribution of CdeC as *Full* pattern was significantly increased in *cotB* spores (65.3 ± 0.9%) compared to wildtype spores (39.7 ± 1.9%) (Figure 5B,D and Figure S5B). In contrast, no significant differences in fluorescence distribution of CdeC-Flag were observed in *cotA* and *CDIF630_02480* spores (Figure 5B). In appendage wildtype spores, only the *Full*, *Edge* and *Bipolar* patterns were present in the parental strain, in a proportion of 40.1%, 37.2% and 4.2%, respectively. An absence of *cotA* led to a significant decrease in *Full* fluorescence pattern to non-fluorescence, while inactivation of *cotB* caused a significant increase in non-fluorescence *Polar* fluorescence patterns and a decrease in spores with *Full* and *Edge* patterns (Figure 5B). The most notorious change in *CDIF630_02480* spores was a decrease and increase in *Edge* and *Polar* patterns, respectively (Figure 5B). Collectively, these results suggest that CdeC is mainly found in the spore surface, forming homogenous round and edge-fluorescence patterns in the presence and absence of appendages, which was mainly affected by the absence of *cotB*.

Finally, the surface distribution of fluorescence intensity of CdeM-FLAG in wildtype spores revealed that in the absence of an appendage, most of the spores lacked fluorescence (60%) (Figure 5B). CdeM-FLAG fusion seems to be mainly found along the spore in the *Full* pattern (25.0 ± 7.6%), but small percentages of spores also showed *Polar* (10.0 ± 2.3%), *Bipolar* (1.0 ± 0.6%) and *Dotted* (3.7 ± 0.7%) patterns. The absence of *cotA* and CDIF630_02480 had no major effect on this pattern (Figure 5B). In contrast, an absence of *cotB* led to a shift from a lack of fluorescence to *Full*, *Edge* and *Polar* fluorescent patterns (Figure 5B). In the presence of an appendage, the most abundant CdeM-fluorescence patterns included non-fluorescence (40.4 ± 17.2%), *Full* (25.6 ± 15.4%) and *Polar* (24.9%) (Figure 5B). Inactivation of *cotA* and *CDIF630_02480* led to a shift in the fluorescence patterns, mainly towards *Full* (Figure 5B), while inactivation of *cotB* shifted the fluorescence towards Full and Polar patterns (Figure 5B). Collectively, these results suggest that in non-appendage spores, an absence of *cotB* increases CdeM-fluorescent spores, while in appendage-positive spores, the absence of all three spore coat proteins led to an increase in CdeM fluorescence.

### 3.7. Effect of Insertional Inactivation of Spore Coat on the Immunofluorescence Distribution of Collagen-like Exosporium Flag Fusion Proteins in the Presence/Absence of Appendage

**Figure 6:**
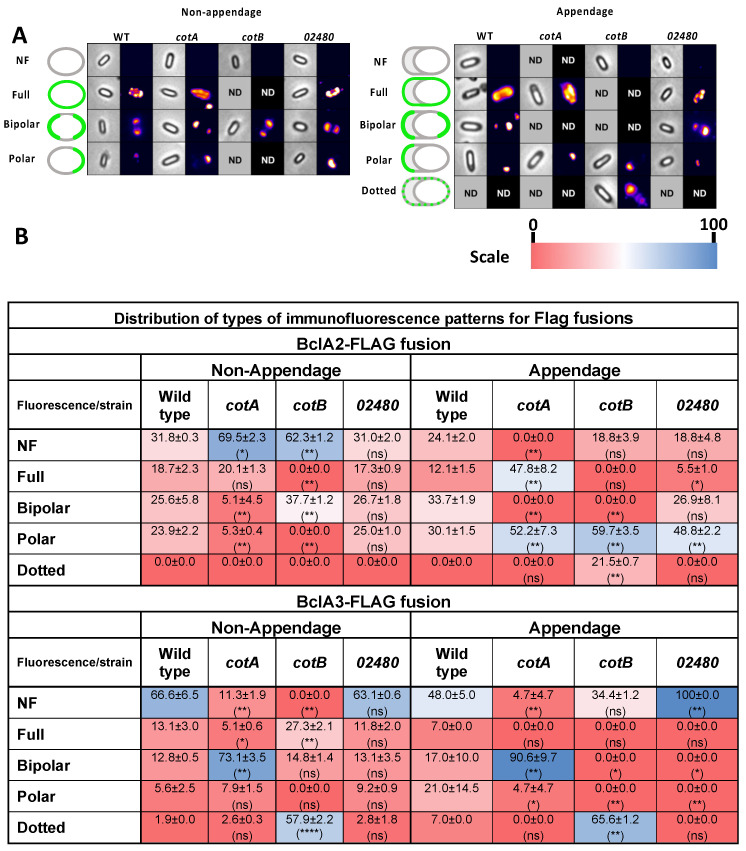
**Effect of absence of spore coat proteins in accessible immunofluorescence of collagen-like BclA-Flag fusions.** (**A**) Representative image of the distribution of fluorescent distribution pattern of BclA2-FLAG on the surface of non-appendage or appendage spores of Wildtype and *cotA (cotA::CT555a*), *cotB* (*cotB::CT329a*) and *CDIF630_02480* (*CDIF630_02480::CT90a*) mutant strains. (**B**) Quantification of fluorescence patterns in non-appendage or appendage spores of wildtype *cotA*, *cotB or cotCB* strains which were first binned by presence or absence of appendage through phase contrast microscopy prior to fluorescence analysis. Data represent the mean and error bars represent the standard error of the mean. Asterisks denote statistical difference with respect to wildtype (ANOVA and Bonferroni multiple comparison test), where * *p* < 0.05, ** *p* < 0.01, **** *p* < 0.0001, and n.s., denotes no significant difference. Heat map depicts the percentage of total of the fluorescence distribution from 0 to 100%.

Analysis of the fluorescence distribution patterns in non-appendage spores carrying BclA2-FLAG fusion revealed similar fluorescence patterns (i.e., *NF*, *Full*, *Bipolar* and *Polar*) and relative abundances as in appendage-positive spores (Figure 6B). The absence of *cotA* and *cotB* increased the abundance of spores that lacked fluorescence, whereas the absence of CotCM did not affect the fluorescence patterns observed in wildtype spores (Figure 6B). In the presence of polar-appendage in wildtype spores, the fluorescence distribution of the collagen-like BclA2-FLAG fusion revealed that some spores had no fluorescence (24.1 ± 2.0%), or *Full* (12.21 ± 1.5%), *Bipolar* (33.7 ± 1.9%) or *Polar* (30.1 ± 1.5%) fluorescence pattern (Figure 6B). *cotA* spores exhibited an increase in the *Full* fluorescence pattern (47.8 ± 8.2%), whereas in *cotB*, the fluorescence patterns shifted towards Polar and Dotted (Figure 6B). The absence of CDIF630_02480 led to a significant increase in the Polar fluorescence pattern (Figure 6B). These results suggest that BclA2-specific fluorescence is primarily distributed, regardless of the presence/absence of the polar appendage, in one or two of the spore-poles, and that *cotA* and *cotB* contribute to this distribution.

Next, we addressed how the absence of spore coat proteins affected the fluorescence distribution of BclA3. In non-appendage wildtype spores, BclA3-FLAG fusion fluorescence was found as *Full* (13.1 ± 3.0%), *Bipolar* (12.8 ± 0.5%), *Polar* (5.6 ± 2.5%) and *Dotted* (1.9 ± 0.0%) patterns (Figure 6B and Figure S6). In non-appendage spores, the absence of *cotA* led to an increase in *Bipolar* pattern, whereas inactivation of *cotB* caused an increase in *Dotted* pattern; no significant changes were observed in the absence of CDIF630_02480 (Figure 6B and Figure S6). In wildtype spores with appendages, BclA3-FLAG fusion fluorescence patterns ranged from non-fluorescence (48.0 ± 5.0%), *Full* (7%), *Bipolar* (17.0 ± 10.0%), *Polar* (21.0 ± 14.5%) and *Dotted* (7%) (Figure 6B and Figure S6). The absence of *cotA* led the majority of the spores to shift to a *Bipolar* fluorescence pattern (Figure 6B and Figure S6), whereas the absence of *cotB* led to a significant increase in the nonfluorescent- and *Dotted*-fluorescent spores (Figure 6B and Figure S6). Notably, in the absence of CotCB, a complete lack of BclA3-specific fluorescence was evidenced in all analyzed spores (Figure 6B and Figure S6). Collectively, these results demonstrate that regardless of the presence or absence of the appendage, most spores lack BclA3 fluorescence, which is likely due to masking by *cotA* and *cotB*.

## 4. Discussion

**Figure 7:**
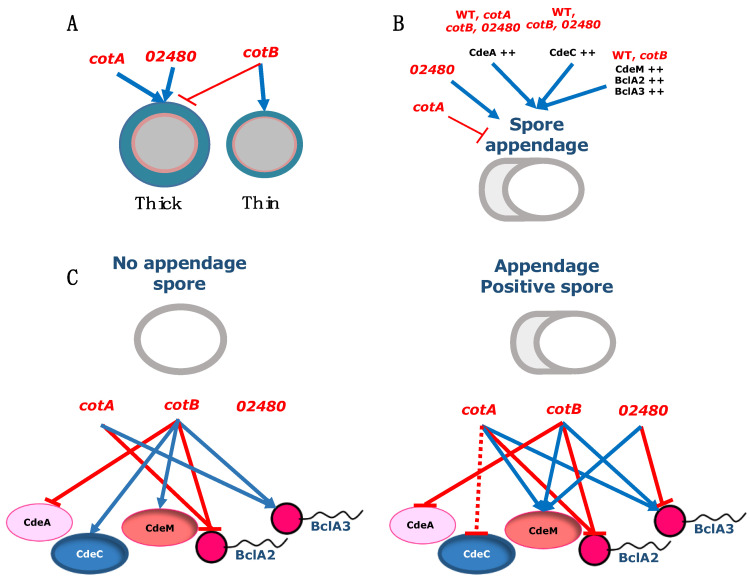
**Schematic representation of the impact of inactivation of spore coat genes and multicopy expression of selected exosporium proteins on relevant spore phenotypes**. (**A**) *cotB* contributes to the formation of thick-exosporium spores, while CDIF630_02480 negatively regulated the thickness of the electron-dense material in thick-exosporium spores, derived from results in Figure 2. (**B**) The formation of spores with a polar appendage is positively and negatively regulated by the spore coat proteins, *cotA* and CDIF630_02480, respectively, while the exosporium cysteine-rich (CdeA, CdeC and CdeM) and the collagen-like BclA2 proteins positively contribute to the formation of appendage-positive spores derived from results in Figure 3. (**C**) Representation of the association between the spore coat proteins *cotA*, *cotB* and CDIF630_02480 on the surface accessibility to anti-flag antibodies of the exosporium proteins CdeA, CdeC, CdeM, BclA2 and BclA3 derived from results of Figure 5 and Figure 6. Blue arrows denote a positive association; dotted blue arrows denote a slight positive association; and red arrows denote a negative association.

*C. difficile* spores are essential for disease initiation and recurrence [5,6,45]. The exosporium layer is essential for their interaction with host surfaces and disease, and has been shown to play a role in the pathogenesis of *C. difficile* infection [7,8,20,44,46,47]. The exosporium layer can be present in *C. difficile* spores as a thick- or a thin-exosporium layer, which differs in the thickness of the electron-dense layer, and its thickness seems to depend on the exosporium morphogenetic protein CdeC. Previous studies in strain 630 demonstrated that both exosporium morphogenetic proteins, CdeC and CdeM, are required for normal levels of the spore coat proteins *cotA* and *cotB* [20]. Prior work included functional analysis of the spore coat proteins *cotA* and *cotB* [25]; however, a thorough analysis of the impact of the absence of these proteins in the spore ultrastructure, appendage formation and exosporium proteins has not been assessed. Consequently, in this work, we have expanded our understanding of the assembly of the outermost layers in *C. difficile* spores (i.e., spore coat and exosporium), by investigating how the absence of three spore coat proteins (*cotA* and *cotB*) and increased expression of selected exosporium constituents affects several spore properties. This work also addresses the role of a previously uncharacterized spore surface protein, CDIF630_02480, on the assembly of *C. difficile* spore surface layers. The results and limitations of this work are discussed, contextualized and summarized (Figure 7).

A primary conclusion of this work is that although the spore coat proteins *cotA*, *cotB* and CDIF630_02480 have a slight impact on several spore structural properties in strain 630, these proteins are not morphogenetic proteins essential for the proper assembly of the spore coat. These observations, at least for *cotB* and CDIF630_02480, are in agreement with prior work by Permpoonpattana et al. (2013); however, the most controversial observation of our TEM results is that here, we provide evidence that the *cotA*::CT555a mutant forms spores with a similar overall ultrastructure to the parental wildtype 630 strain (Figure 2A), contrasting with prior work by Permpoonpattana et al. (2013) that demonstrated that this same mutant strain led to the formation of two distinctive spore morphotypes; ill-formed *cotA* spores and wildtype looking *cotA*-spores, creating a bi-population of spores, where ill-formed spores misassembled the outermost spore layers (i.e., spore coat and exosporium) [25]. The main difference between our current and prior work relates to the conditions employed in spore preparation; in this work, we employed 70:30 agar plates for spore production compared with liquid BHIS media employed in Permpoonpattana et al. (2013). Preparation of *B. subtilis* spores in solid medium leads to a higher degree of crosslinking in the outer layers relative to liquid medium [48]. Thus, this higher rigidity of *cotA* spores prepared in solid medium might be masking the role of *cotA* in the morphogenesis of the spore surface layers. Although we have previously observed that *C. difficile* spores of strain R20291 prepared in solid media versus liquid conditions have a similar ultrastructure [17], this statement might not be the case for a 630 genetic background, for which we are currently revising the role of *cotA* in spore coat and exosporium assembly in the epidemically relevant R20291 strain.

Another relevant conclusion of this work is that *cotB* was associated with the formation of thick-exosporium spores, as its absence led *cotB* spores to produce less thick-exosporium spores (Figure 2A–C and Figure 6A). The potential role of *cotB* in the formation of thick-exosporium spores is in accordance with *cotB* being part of the spore coat and interface with the exosporium layer [14]. In prior work concerning strain 630, we have functionally characterized the exosporium morphogenetic proteins CdeC and CdeM, demonstrating that while CdeC has a role in spore coat and exosporium assembly, CdeM is essential only for the assembly of the exosporium layer [20]. However, overexpression of CdeC, but not CdeM, leads to an increased proportion of thick-exosporium spores and increased thickness, suggesting that CdeC may be the driver in exosporium assembly. The link between *cotB* and CdeC is supported by the genetic dependency that *cotB* exhibits with CdeC, where the abundance of *cotB* in spore coat/exosporium extracts is partially dependent on CdeC [20]. Intriguing questions that arise include whether *cotB* and CdeC directly or indirectly interact during spore assembly, which is a matter of current study in our lab.

This work also contributes to our understanding of how, in thick-exosporium spores, *cotA* and CDIF630_02480 seem to control the thickness of the spore coat and exosporium, respectively (Figure 2A–C and Figure 6A). The fact that spores lacking *cotA* have a thicker coat was unexpected, and this may suggest an auxiliary role of *cotA* in coat assembly. Most notably, this phenotype was only observed in thick-exosporium spores, supporting the hypothesis that the outer layers of both exosporium morphotype spores is governed through different pathways [9]. The only spore coat morphogenetic protein identified to date is CotL, a lysine-rich protein that is unique to the Peptostreptococacceae family and was recently shown to be essential for proper spore coat formation [49]. An absence of CotL leads to an absence of *cotB* in spore coat/exosporium extracts; however, it is unclear whether a genetic dependency between CotL and *cotA* also exists. Further work is warranted to address these questions. Regarding the association of CDIF630_02480 and exosporium thickness, the fact that the absence of CDIF630_02480 led to increased thickness of the exosporum layer in thick-exosporium spores suggests that this spore surface protein might be a negative structural regulator of exosporium thickness. Future work addressing the spatial and temporal expression profile of CDIF630_02480 and how it contributes to spore surface assembly might shed light on the role of this protein conserved uniquely in the Peptostreptococcaceae and Clostridiaceae family members.

The polar appendage of *C. difficile* spores is a structure that is easily evidenced by phase contrast microscopy, allowing single-spore quantification. Phase contrast analysis of *cotA*, *cotB* and *CDIF630_02480* spores demonstrates that *cotA* is positively associated with the presence of appendage-bearing spores (Figure 3 and Figure 7B), whereas CDIF630_02480 is negatively associated with the formation of appendage-positive spores (Figure 3 and Figure 7B), while inactivation of *cotB* had no effect on the abundance of appendage-bearing spores. It was most striking to observe that incorporation into wildtype and the *cot* mutant strains of a multicopy plasmid, of the pMTL series (i.e., pMTL82151), expressing Cde or BclA exosporium proteins under the control of their native promoter led to changes in the abundance of appendage-bearing spores (Figure 3 and Figure 7B). This highly segregationally stable and pMTL82151 plasmid is likely to lead to an increased copy number in strain 630, in a similar manner to the segregationally stable pMTL84151 for strain R20291, where we observed an increased copy number of spore coat and exosporium proteins [16]. Thus, in this work, we observed that increasing the copy number of all three cystine-rich protein-encoding genes, CdeA, CdeC and CdeM as well as the collagen-like BclA2 encoding gene, led to an increase in appendage-bearing spores, regardless of the spore *cot* genetic background, suggesting that they might directly or indirectly be implicated in appendage formation. Studies of strain 630 demonstrated that the polar appendage was at least partially dependent on CdeM, as observed in spores of a insertional CdeM mutant strain [21], which is in agreement with our results that increased copies of CdeM positively affect appendage-bearing spores. Appendage bearing-spores of strains 630 and R20291 can be enriched through a density gradient, and this enrichment is associated with enrichment of thick-exosporium spores in both strains, 630 and R20291 [16,21]. Work investigating strain 630 has shown that wildtype appendage-bearing spores germinate faster than spores containing a shorter or smaller appendage [21]. Further studies to establish the role of the appendage in *C. difficile* spore biology, how it associates to thick-exosporium spores and its contribution to pathogenesis of CDI are warranted.

A final contribution of this work is that by using an antibody accessibility assay, we provide evidence of how Cot A, *cotB* and CDIF630_02480 impact the accessibility of the cysteine-rich Cde and the BclA collagen-like exosporium proteins, and how this accessibility is affected by the presence and absence of the spore appendage. It is worth noting that the contribution of the spore coat proteins *cotA*, *cotB* and CDIF630_02480 to the surface accessibility of all selected exosporium proteins was protein specific and, in some instances, these associations were dependent on the presence of the spore appendage. For example, the surface accessibility of CdeA was only dependent on *cotB* in both spores with and without polar appendage, suggesting that CdeA requires *cotA* for its surface accessibility. Another noteworthy observation was that the surface accessibility of CdeC increased in the absence of *cotB* only in spores lacking appendages (Figure 5 and Figure 7C), which correlates with the genetic dependency between CdeC and *cotB* that we previously reported [20], and with the fact that both proteins seem to be implicated with the formation of thick-exosporium spores, as shown in Figure 1 and in our prior work [16]. Strikingly, the surface accessibility of CdeM exhibited a negative dependency on the spore proteins, and in appendage-negative spores, the absence of *cotB* led to an increase in CdeM-surface accessibility (Figure 5 and Figure 7C), whereas in appendage-bearing spores, CdeM-surface accessibility increased in the absence of all three spore coat proteins (Figure 5 and Figure 7C). This dependency could be attributed to a masking effect of *cotA*, *cotB* and/or CDIF630_02480 impeding antibody binding. In a similar manner, the surface accessibility of the collagen-like proteins BclA2 was also dependent on the spore coat proteins *cotA* and *cotB*, but not CDIF630_02480, independent of the presence and absence of the spore appendage (Figure 6 and Figure 7B). However, in the case of BclA3, we observed that its surface accessibility increased in the absence of *cotA* and *cotB* independently of the presence of the spore appendage, suggesting that, at least in 630 spores, BclA3 might be masked by both *cotA* and *cotB* (Figure 6 and Figure 7B). The fact that the presence of the spore polar appendage indeed affected some of these associations suggests that the assembly mechanisms of the spore coat and exosporium layer differ between spores lacking the polar appendage and appendage-bearing spores. Overall, these observations—while informative and suggestive of potential associations (Figure 7)—have limitations that should be considered. Among these was that given the low amounts of spores harvested from the sporulating cultures, we were unable to quantify how the absence of the spore coat proteins affected the relative abundance of CdeA, CdeC, CdeM, BclA2 and BclA3, which would strengthen the genetic dependencies between spore coat and exosporium constituents, as we have previously shown [20]. In this context, it is unclear if the decrease in fluorescence-signal is attributed to the lack of proteins or a masking effect. Further studies to identify the interaction network and direct protein–protein interactions are currently being conducted in our lab.

This work has some limitations that need to be considered for the proper interpretation of the results being exposed. The spore coat mutant strains *cotA, cotB* and CDIF630_02480 were constructed via insertional inactivation using ClosTron technology at base pair positions 555-, 329- and -90 downstream of the start codon [25], leading to a significant in-frame polypeptide that may retain assembly properties, as we have previously shown for the N-terminal domains of the BclA1, BclA2, BclA3 and CdeC exosporium proteins [15,43], suggesting the need to revisit these studies with complete gene-deletion techniques. Additional limitations of these studies were that we did not conduct proper complementation studies to address whether the phenotypes observed in the *cot*-mutant strains related to formation of thick-exosporium spores, thickening of the exosporium layer and formation of the polar appendage. Our rationale in this regard is that complementation with the corresponding *cot* gene and its native promoter in a multi-copy pMTL plasmid would lead to an increased copy number of the gene of interest, very likely leading to similar perturbations to those observed for the exosporium genes in this work (Figure 3). Indeed, these plasmids lead to an overexpression of the gene of interest, under its native promoter, as demonstrated by others in strain 630 [42]. In work by Permpoonpattana et al. (2013), it appears that the authors constructed inducible complementation plasmids and observed similar levels of *cotA* and *cotB* between the wildtype strains and the complemented strains; however, decoupling complementation (i.e., inducible) from its native promoter distorts the timing when this protein is required. Several tools are available to properly complement with a single copy of the gene of interest, including (i) generating a *pyrE*-mutant derivative followed by *pyrE*-based complementation into the *pyrE* loci, as we and others have reported [8,50]; and (ii) using CRISPR-cas9 into the loci of the gene of interest swapping the intron insertion [51]. Despite these limitations, whole-genome analysis demonstrates that a single intron copy was observed in each mutant with no off-target mutations, suggesting that the phenotypes observed are solely due to the disruption of the *cotA*, *cotB* and the *CDIF630_02480* gene.

Another limitation of this work is the use of the genetically manipulatable *C. difficile* strain 630, which forms spores with a smooth electron-dense exosporium layer that lacks the hair-like projections commonly observed in most clinically relevant strains [5,17,18]. Therefore, these results will need to be validated in an epidemically relevant strain that produces an exosporium layer similar to most clinically relevant strains [7,17], such as strain R20291. Overall, this work contributes by providing further description of the high variability that has been observed in *C. difficile* spores in recent studies [16,17,18], and underscores the need to refine our tools to dissect the mechanisms underlying this degree of heterogeneity. Further studies to directly demonstrate these interactions and the interconnections that underly the assembly of *C. difficile* spore coat and exosporium layers are warranted.

## Figures and Tables

**Table 1:** Bacterial strains and plasmids used.

Strain or Plasmid	Relevant Characteristic	Source/Reference
*C. difficile*		
630Δ*ermB*	An erythromycin-sensitive derivative of *C. difficile* strain 630	[14]
630Δ*ermB*(pDP345)	630∆*ermB* carrying *cdeC*-FLAG fusion in pMTL	[14]
630Δ*ermB*(pDP360)	630∆*ermB* carrying *cdeM*-FLAG fusion	[14]
630Δ*ermB*(pDP363)	630∆*ermB* carrying *bclA3*-FLAG fusion	[14]
630Δ*ermB*(pDP365)	630∆*ermB* carrying *cdeA*-FLAG fusion	[14]
630Δ*ermB*(pDP369)	630∆*ermB* carrying *bclA2*-FLAG fusion	[14]
630Δ*ermB cotA*	630 Δ*ermB cotA*::intron *ermB*	[25]
630Δ*ermB cotA*(pDP345)	630∆*ermB cotA::*intron carrying *cdeC*-FLAG fusion	This work
630Δ*ermB cotA* (pDP360)	630∆*ermB cotA::*intron carrying *cdeM*-FLAG fusion	This work
630Δ*ermB cotA* (pDP363)	630∆*ermB cotA::*intron carrying *bclA3*-FLAG fusion	This work
630Δ*ermB cotA* (pDP369)	630∆*ermB cotA::*intron carrying *bclA2*-FLAG fusion	This work
630Δ*ermB cotA* (pDP365)	630∆*ermB cotA::*intron carrying *cdeA*-FLAG fusion	This work
630Δ*ermB cotB*	630 Δ*ermB cotB*::intron *ermB*	[20,25]
630Δ*ermB cotB* (pDP345)	630∆*ermB cotB::*intron carrying *cdeC*-FLAG fusion	This work
630Δ*ermB cotB* (pDP360)	630∆*ermB cotB::*intron carrying *cdeM*-FLAG fusion	This work
630Δ*ermB cotB* (pDP361)	630∆*ermB cotB::*intron carrying *bclA1*-FLAG fusion	This work
630Δ*ermB cotB* (pDP363)	630∆*ermB cotB::*intron carrying *bclA3*-FLAG fusion	This work
630Δ*ermB cotB* (pDP369)	630∆*ermB cotB::*intron carrying *bclA2*-FLAG fusion	This work
630Δ*ermB cotB* (pDP365)	630∆*ermB cotB::*intron carrying *cdeA*-FLAG fusion	This work
630Δ*ermB CDIF630_02480*	630 Δ*ermB CDIF630_02480*::intron *ermB;* 630 strain reference genome CP010905.2 (CD2245.1 of the strain 630 reference genome AM180355.1).	[25] and This work.
630Δ*ermB CDIF630_02480* (pDP345)	630∆*ermB CDIF630_02480::*intron carrying *cdeC*-FLAG fusion	This work
630Δ*ermB CDIF630_02480* (pDP360)	630∆*ermB CDIF630_02480::*intron carrying *cdeM*-FLAG fusion	This work
630Δ*ermB CDIF630_02480* (pDP361)	630∆*ermB CDIF630_02480::*intron carrying *bclA1*-FLAG fusion	This work
630Δ*ermB CDIF630_02480* (pDP363)	630∆*ermB CDIF630_02480::*intron carrying *bclA3*-FLAG fusion	This work
630Δ*ermB CDIF630_02480* (pDP369)	630∆*ermB CDIF630_02480::*intron carrying *bclA2*-FLAG fusion	This work
630Δ*ermB CDIF630_02480* (pDP365)	630∆*ermB CDIF630_02480::*intron carrying *cdeA*-FLAG fusion	This work
*Plasmids*		
pDP345	pMTL82151 carrying *cdeC*_630_ labeled at the C-terminal with FLAG as a reporter tag in NdeI/HindIII sites.	[19]
pDP360	pMTL82151 carrying *cdeM*_630_ labeled at the C-terminal with FLAG as a reporter tag in KpnI/SalI sites.	[14]
pDP363	pMTL82151 carrying *bclA3*_630_ labeled at the C-terminal with FLAG as a reporter tag in KpnI/SalI sites.	[14]
pDP365	pMTL82151 carrying *cdeA*_630_ labeled at the C-terminal with FLAG as a reporter tag in KpnI/SalI sites.	[14]
pDP369	pMTL82151 carrying *bclA2*_630_ labeled at the C-terminal with FLAG as a reporter tag in KpnI/SalI sites.	[14]

## Data Availability

Whole genome sequencing reads are available under SRA PRJNA875441 at the NCBI data base.

## Supplementary Materials

The following supporting information can be downloaded at: https://www.mdpi.com/article/10.3390/microorganisms10101918/s1, Supplementary Figure S1. Pairwise alignment of the *cotA* loci of *C. difficile* 630 delta *ermB* wild-type strain (cotA wt allele) and *cotA::CT* mutant strain (cotA::CT allele). Supplementary Figure S2. Pairwise alignment of the *cotB* loci of *C. difficile* 630 delta *ermB* wild-type strain (cotB wt allele) and *cotB::CT* mutant strain (cotB::CT allele). Supplementary Figure S3. Pairwise alignment of the *CD2480* loci of *C. difficile* 630 delta *ermB* wild-type strain (CD2480 wt allele) and *CDIF630_02480::CT* mutant strain (*CDIF630_02480::CT* allele). Supplementary Figure S4. Effect of absence of spore coat proteins and over-expression of exosporium proteins in the formation of polar appendage in *C. difficile* spores. Effect of over expression of exosporium proteins in appendage formation in wild-type, *cotA::CT555a*, *cotB::CT329a* and *CDIF630_02480::CT90a* strains. Supplementary Figure S5. Distribution of fluorescent pattern of CdeC-FLAG (A) and CdeM-FLAG (B) on thesurface of purified spores of Wild type and *cotA* (*cotA::CT555a*), *cotB* (*cotB::CT329a*) and *CDIF630_02480* (*CDIF630_02480::CT90a*) mutant strains by immunofluorescence. Supplementary Figure S6. Distribution of fluorescent pattern of BclA3-FLAG on the surface of purified spores of Wild type and *cotA* (*cotA::CT555a*), *cotB* (*cotB::CT329a*) and *CDIF630_02480* (*CDIF630_02480::CT90a*) mutant strains by immunofluorescence.
