# Supplementary material for: Role of the Spore Coat Proteins CotA and CotB, and the Spore Surface Protein CDIF630_02480, on the Surface Distribution of Exosporium Proteins in Clostridioides difficile 630 Spores
Source: Microorganisms. 2022 Sep 27;10(10):1918. doi: 10.3390/microorganisms10101918 (PMC9610443; doi:10.3390/microorganisms10101918)
Supplement: Supplementary file 1 [file microorganisms-10-01918-s001.zip › microorganisms-1827605-supplementary.pdf]

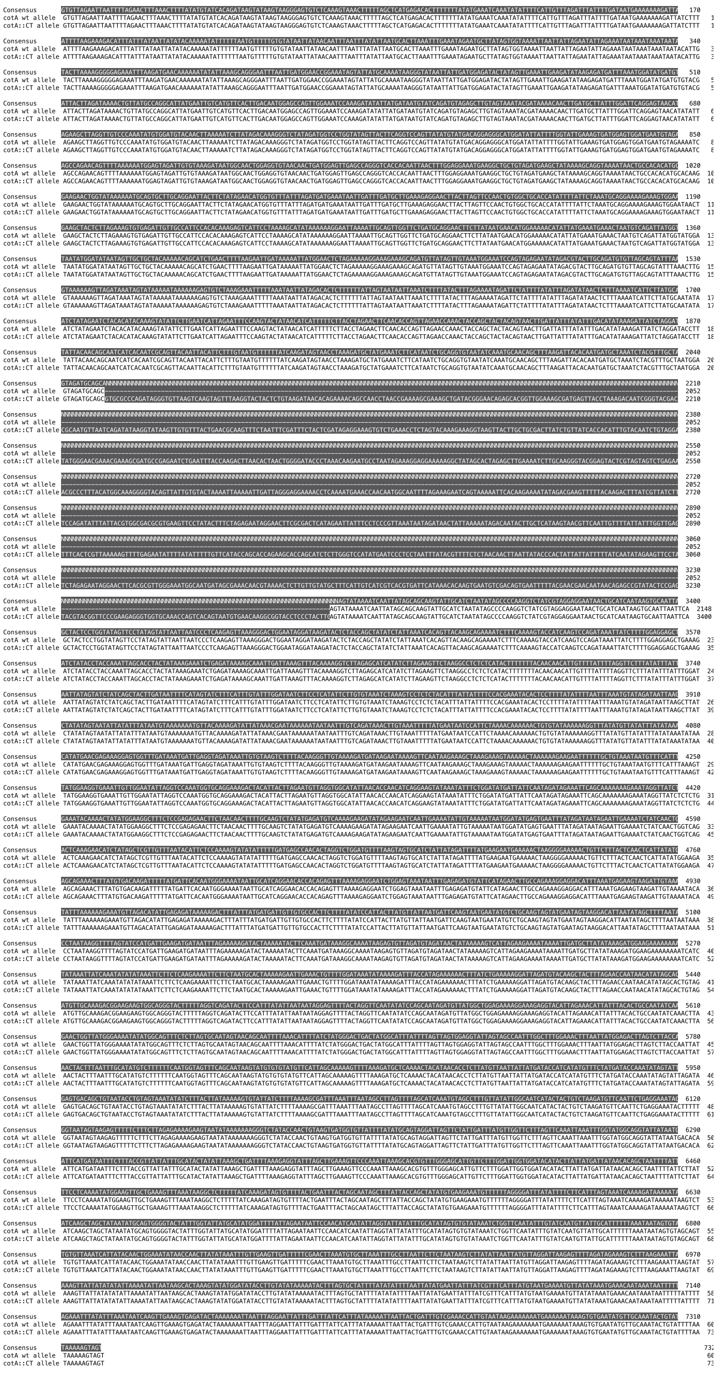

Supplementary Figure S1. Pairwise alignment of the *cotA* loci of *C. difficile* 630 delta *ermB* wild-type strain (cotA wt allele) and *cotA::CT* mutant strain (cotA::CT allele). Both wild-type and mutant alleles were obtained from *de novo* assembly of illumine reads with SPAdes. Contigs were annotated against reference NCBI database, and the contigs containing the alleles of interest were pulled out and used for pairwise alignment using the in-house built alignment tool. N, indicates no consensus between both alleles.

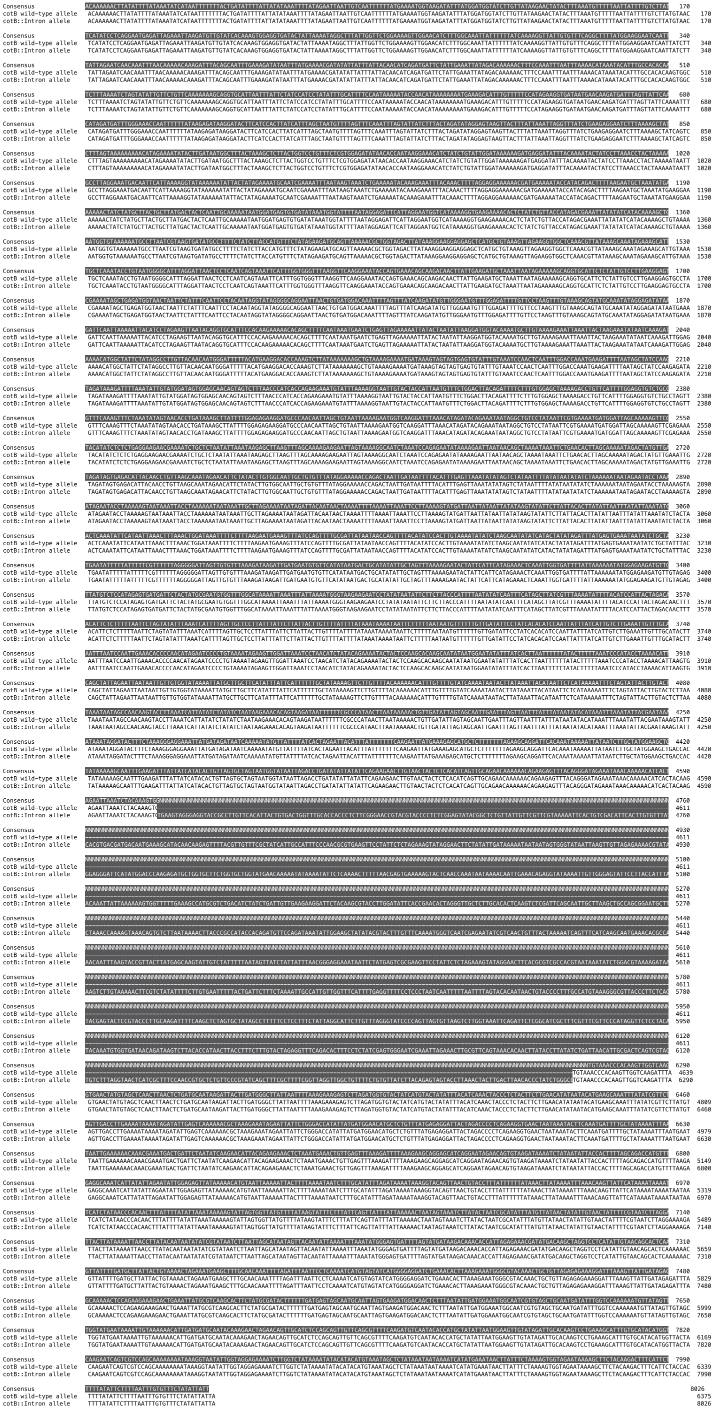

Supplementary Figure S2. Pairwise alignment of the *cotB* loci of *C. difficile* 630 delta *ermB* wild-type strain (cotB wt allele) and *cotB::CT* mutant strain (cotB::CT allele). Both wild-type and mutant alleles were obtained from *de novo* assembly of illumine reads with SPAdes. Contigs were annotated against reference NCBI database, and the contigs containing the alleles of interest were pulled out and used for pairwise alignment using the in-house built alignment tool. N, indicates no consensus between both alleles.

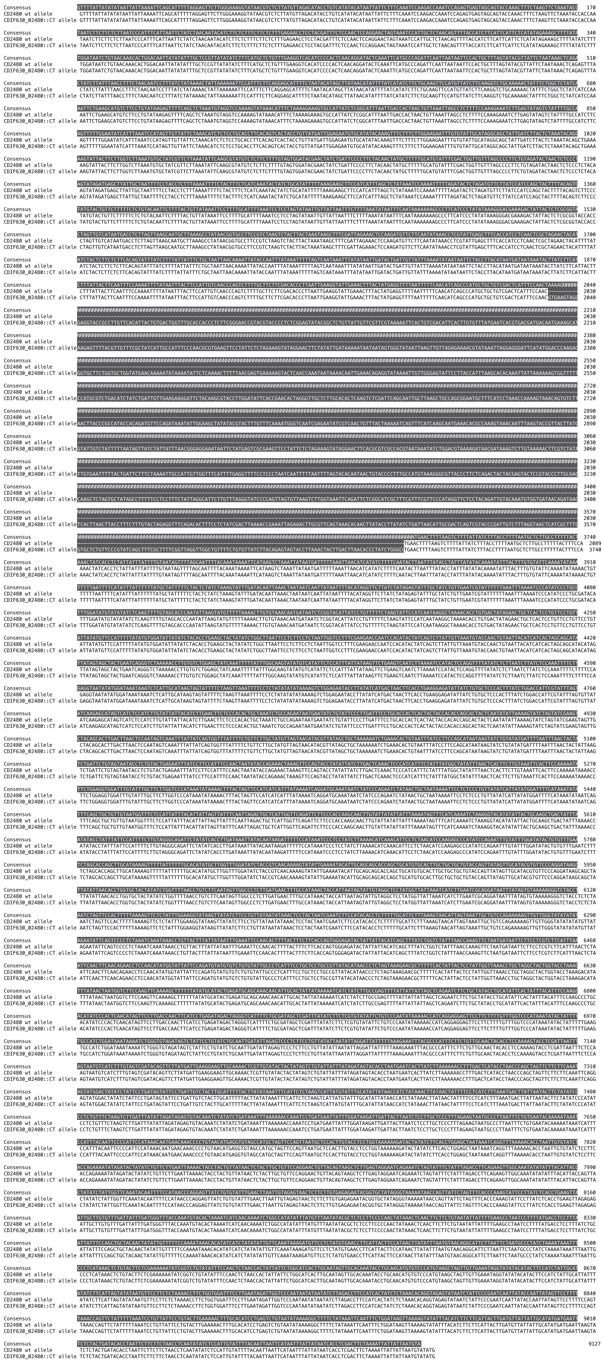

Supplementary Figure S3. Pairwise alignment of the *CD2480* loci of *C. difficile* 630 delta *ermB* wild-type strain (CD2480 wt allele) and *CDIF630_02480::CT* mutant strain (*CDIF630_02480* ::CT allele). Both wild-type and mutant alleles were obtained from *de novo* assembly of illumine reads with SPAdes. Contigs were annotated against reference NCBI database, and the contigs containing the alleles of interest were pulled out and used for pairwise alignment using the in-house built alignment tool. N, indicates no consensus between both alleles.

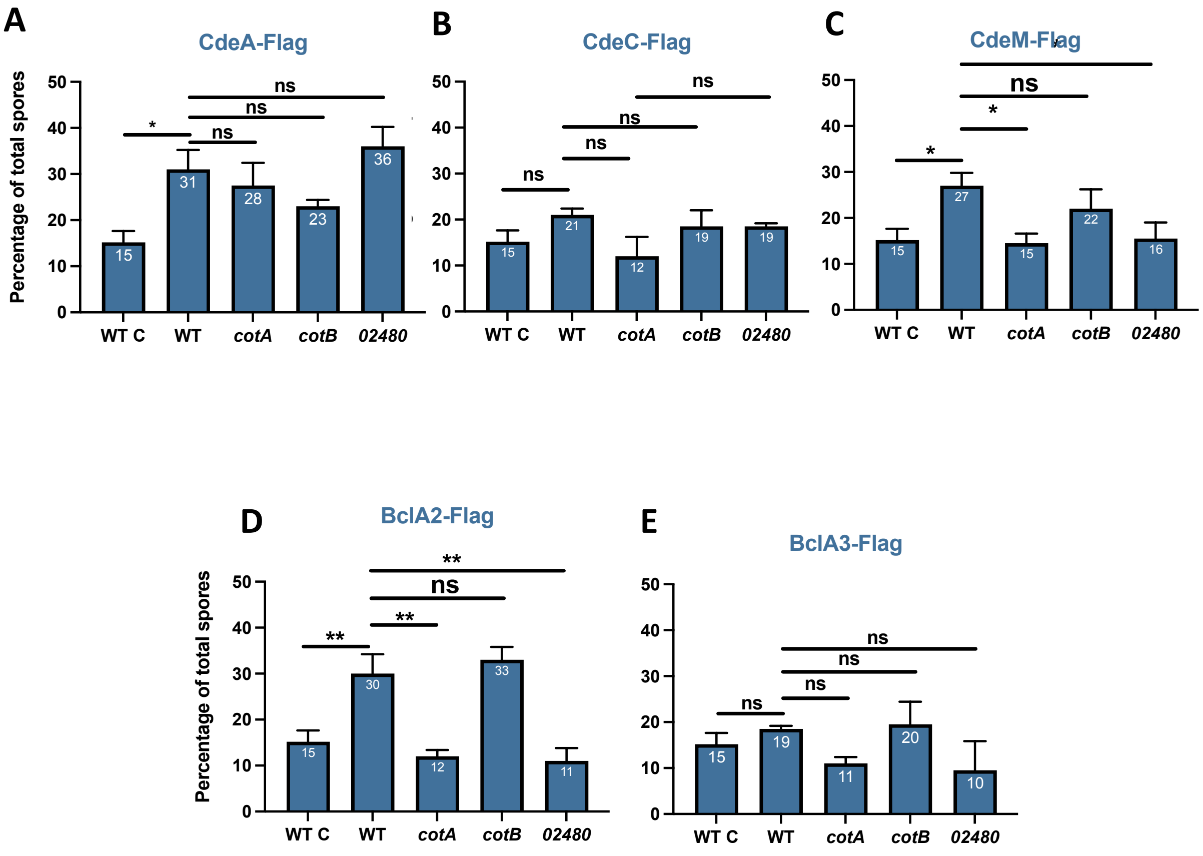

Supplementary Figure S4. Effect of absence of spore coat proteins and over-expression of exosporium proteins in the formation of polar appendage in *C. difficile* spores. Effect of over expression of exosporium proteins in appendage formation in wild-type, *cotA::CT555a*, *cotB::CT329a* and *CDIF630_02480::CT90a* strains. Purified spores where analyzed by phase-contrast microscopy, as described in the method section. *C. difficile* strains carrying plasmid pMTL82151 with CdeA-Flag (A), CdeC-Flag (B), CdeM-Flag (C), BclA2-Flag (D) and BclA3-Flag (E) fusions. The spores with an appendage in each strain's spore population were analyzed by a phase-contrast micrograph where at least 800 spores were quantified. Asterisks denote statistically significant differences, * $p >$ 0.01; ns, not significant (One-way ANOVA and Sidak´s multiple comparison test).

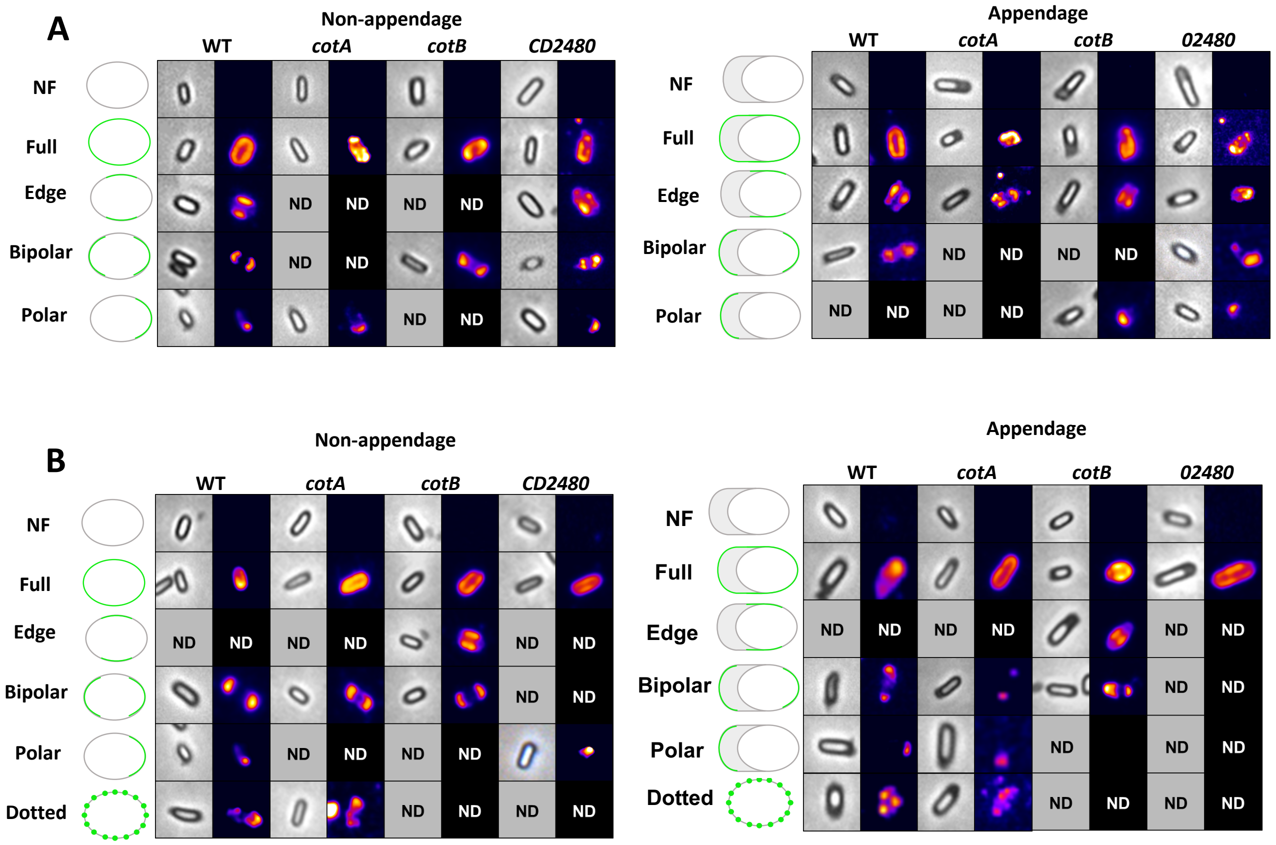

Supplementary Figure S5. (**A,B**) Distribution of fluorescent pattern of CdeC-FLAG (A) and CdeM-FLAG (B) on the surface of purified spores of Wild type and *cotA* (*cotA::CT555a*), *cotB* (*cotB::CT329a*) and *CDIF630_02480* (*CDIF630_02480::CT90a*) mutant strains by immunofluorescence. Data are representative micrographs of three independent experiments.

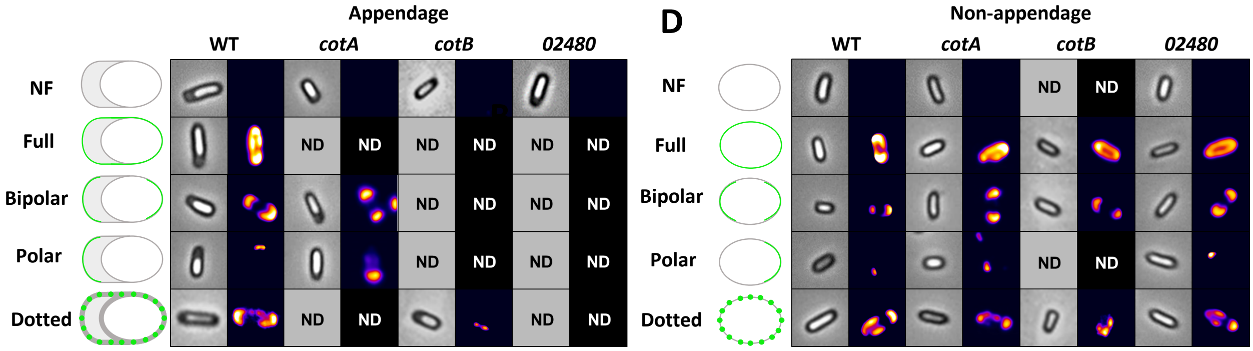

Supplementary Figure S6. Distribution of fluorescent pattern of BclA3-FLAG on the surface of purified spores of Wild type and *cotA (cotA::CT555a)*, *cotB* (*cotB::CT329a*) and *CDIF630_02480* (*CDIF630_02480::CT90a*) mutant strains by immunofluorescence. Data are representative micrographs of three independent experiments.
